# Organic Photovoltaic Materials for Solar Fuel Applications: A Perfect Match?

**DOI:** 10.1021/acs.chemmater.3c02286

**Published:** 2024-02-15

**Authors:** Catherine M. Aitchison, Iain McCulloch

**Affiliations:** Department of Chemistry, University of Oxford, 12 Mansfield Road, Oxford OX1 3TA, United Kingdom

## Abstract

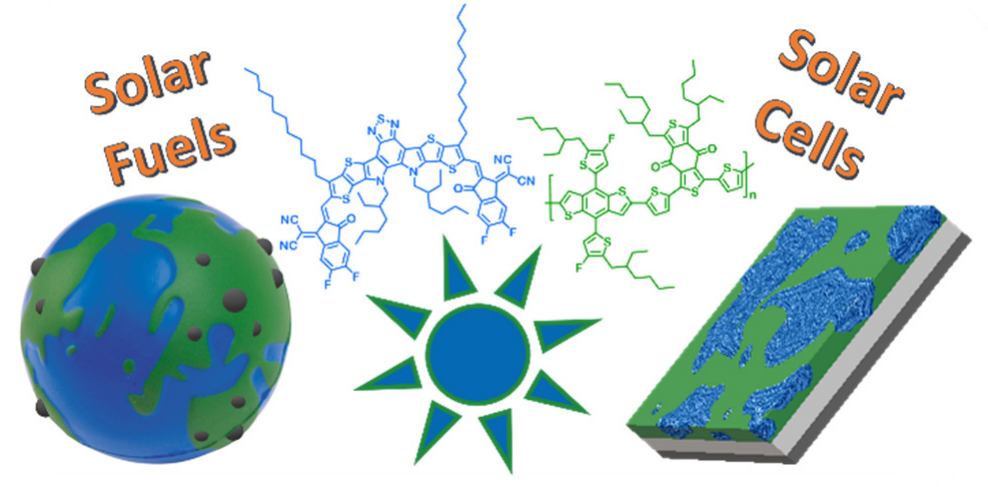

This work discusses the use of donor and acceptor materials from organic photovoltaics in solar fuel applications. These two routes to solar energy conversion have many shared materials design parameters, and in recent years there has been increasing overlap of the molecules and polymers used in each. Here, we examine whether this is a good approach, where knowledge can be translated, and where further consideration to molecular design is required.

The idea of converting light into usable energy is not a new one. In a 1912 *Science* article, Ciamician asked presciently “*Is fossil solar energy the only one that may be used in modern life and civilization?*”.^1^ In 2018, the capacity for electricity derived from photovoltaics (PV) had reached 505 GW.^2^ Advances in efficiency and scale-up of production have allowed the price of solar electricity to become competitive with fossil fuel-derived electricity.^3^ Even replacing existing coal plants with new PV is becoming increasingly cost effective.^4^ These advances mean that PV technology is on track to meet a “Sustainable Development Scenario” consistent with the Paris climate agreement.^5,6^ However solar electrcity, or indeed any renewable electricity, alone cannot meet the complex energy requirements of modern society; heating buildings, transport, and industrial processes make up 60% of energy demand^7,8^ and mostly use fuel-based energy, rather than electrical energy.^8,9^ The United States, United Kingdom, European Union, and International Criminal Court have all stated that hydrogen will be an important replacement to fossil fuels in these systems. Reports analyzing the technoeconomics of solar fuel production have indicated that photocatalytic (PC) water splitting, could provide the cheapest route to renewable hydrogen production.^10,11^

Organic semiconductors are less established than their inorganic counterparts for both PV and PC applications but offer an interesting alternative, as they are comprised of cheap, easily modifiable aromatic units with huge combinatorial flexibility.^12^ While organic photovoltaics (OPV) have been widely studied since the early 2000s, organic materials for solar fuel applications have only become a significant area of investigation in the past decade. Despite this late start, research in organic photocatalysts for hydrogen production is booming. Google Scholar metrics searching for “organic” + “photocatalytic hydrogen” have now surpassed “organic photovoltaics” in terms of articles per year (Figure 1).

**Figure 1:**
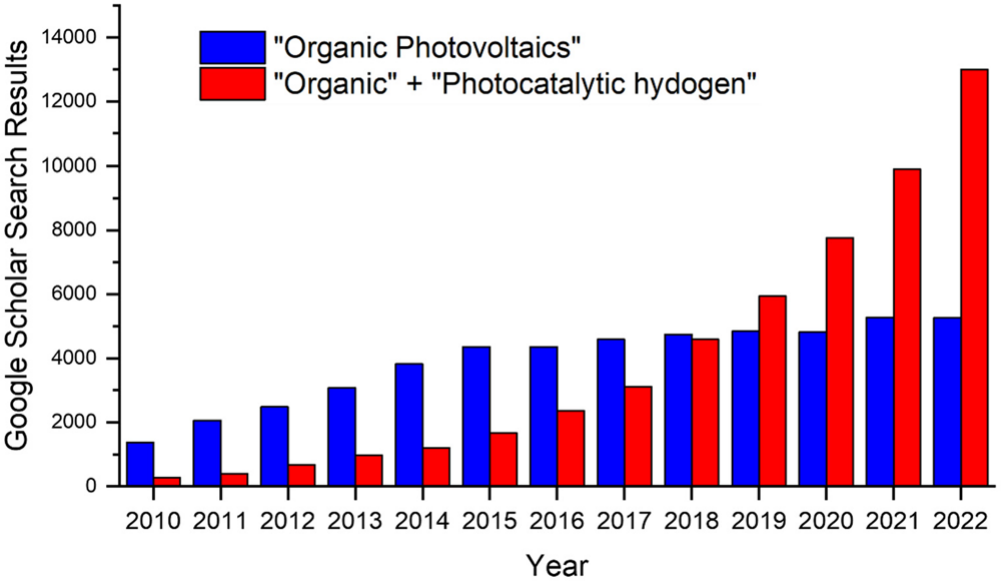
Google Scholar search results for “Organic Photovoltaics” versus “Organic” + “Photocatalytic Hydrogen” when filtering for individual years.

Organic photovoltaics has cumulatively provided a huge library of materials—sometimes just sitting on lab shelves. Synthetic research chemists and commercial partners have frequently put huge effort into optimizing their synthesis, often with considerable effort to also use cheap, eco-friendly, or sustainable choices. The question, however, is should the solar fuels community simply adopt these materials and use without further modifications or are there nuances in the performance of photocatalysts that require further molecular design optimizations.

## Material Requirements for Solar Fuel Photocatalysis

If one considers the steps involved in photovoltaics and photocatalysis then the materials properties required for each become clear. Both applications require the photoexcitation of a semiconductor, diffusion of excitons to an interface where separation into free charge carriers can occur, and then the efficient transportation of these charges to the material surface. Here, however, the material requirements diverge; in PV, charges are extracted by electrodes (normally through electron or hole transport layers). In PC, either charges are transferred directly from the semiconductor to redox reagents or a metal cocatalyst is used to facilitate this charge transfer. Hence, for a particular material property, a number of factors should be considered.

### Thermodynamics

The optical gap of the semiconductors is highly important for both applications to maximize *absorption of solar irradiation*. Similarly, the *energetic offset of donor and acceptor* semiconductor energy levels in a heterojunction is important, for PV and for PC, to enable the efficient separation of excitons into charges. Exciton binding energies in state-of-the-art OPV materials are approximately 0.1–0.3 eV, and donor/acceptor ionization potential (IP) offsets of 0.5 eV, along with smaller electron affinity (EA) offsets, are generally considered optimal to drive charge separation without significant loss of potential.^13^ PV also benefits from a ∼1 eV donor HOMO, acceptor LUMO gap to provide a reasonable *V*_OC_, but the *absolute* energy levels of the materials are less significant.

In PC, the absolute energy levels of the frontier molecular orbitals are crucial. At the very least the donor IP and acceptor EA must straddle the proton reduction potential and the relevant oxidation potential at the pH and solvent conditions used. The optimal thermodynamic driving force for each half reaction is a difficult parameter to define. FMO energies are often used as a proxy, but it is the energy of polarons, including losses associated with their transport to the active site, that dictates the “overpotential” each material possesses. Many materials are active for proton reduction with reported overpotentials of less than 0.3 eV,^14,15^ but some photocatalysts ascribe their high activity to very large >1 eV overpotentials, particularly for water oxidation.^16^ Driving force considerations are also complicated by the fact that reporting of organic semiconductor energies is often inconsistent, with processing and environmental effects playing a role in their measured values, as well as variable conversion between electrochemical potentials calculated to SHE and FMO energies measured relative to vacuum.^17^

Ultimately the driving force that would give the best photocatalytic activity will vary depending on the rate-limiting steps for each system. The most active overall water splitting (OWS) materials currently have band gaps of around 2 eV. The donor IP to acceptor EA energy gap for OPV materials is typically significantly less than this, which could limit their application in single-photocatalyst OWS. OPV materials should, however, be ideally suited for Z-schemes. These employ separate hydrogen evolution photocatalysts (HEPs) and oxygen evolution photocatalysts (OEPs) coupled by an electron mediator or a reversible solution-based redox shuttle.^18^ The latter of these also has the advantage of enabling spatially separated hydrogen and oxygen production, thus removing the possibility of hydrogen and oxygen recombination. It should be noted that the rate of this back reaction can also be reduced by optimizing the reaction temperature^19^ and through cocatalyst design.^20^

**Figure 2:**
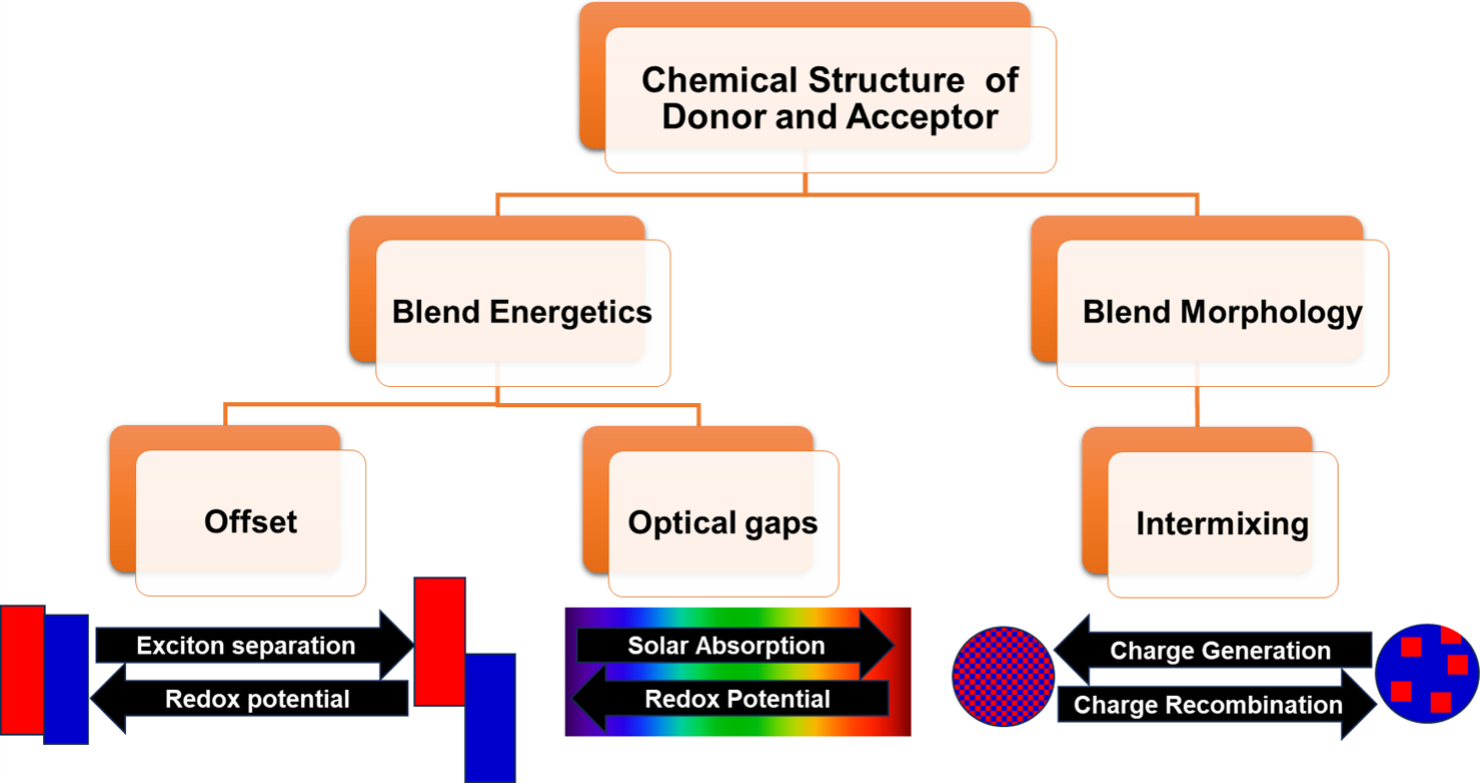
Cartoon showing the relationship between chemical design and some of the factors that determine photocatalytic activity.

OPV, organic PC, and indeed the whole field of organic electronics can utilize molecular design to tune energy levels.^12^ Generalized molecular design strategies are not the remit of this discussion but typically involve iterative variation of specific functional groups and can give large changes to energy, conformation, and packing by changing just one bond or atom. Altered properties may lead to improved metrics for one of the aforementioned factors but will frequently hinder another. Figure 2 illustrates some such factors. The challenge for materials design is to balance these. The weighting to each factor must consider the bottlenecks in the photocatalytic process and thus must be viewed from a kinetic as well as thermodynamic viewpoint.

### Kinetics

Following photon absorption, the initial excited state processes are very similar in OPV and organic PC. Sufficient exciton diffusion lengths—a function of both lifetime and mobility—are required to get excitons from the point of excitation to the donor:acceptor charge separation interface. *Exciton diffusion lengths* are affected by the conformation of the monomer units, the distances between, and the relative orientation of molecules or chains (packing). In general, lifetime and mobility are increased by more ordered systems with fewer defects that could encourage recombination. Such properties may be affected by the molecular structure or chain length, but processing methods can also govern the degree of crystallinity and the particular stacking mode present in a material. *Charge carrier lifetime and mobility* are similarly important for transport of hole polarons (on the donor) and electron polarons (on the acceptor) to the active site. Similarly to exciton diffusion, these properties are governed by a complicated combination of backbone conformation and intermolecular packing, as well as factors such as polarity and reorganization energies.

The distances across which excitons need to diffuse to be productive is determined by the *blend morphology*. Increased intermixing between donor and acceptor phases decreases the distance an exciton must travel to reach an interface and so increases the probability of charge generation. However, increasing the phase separation between the donor and the acceptor—up to the limit of a bilayer structure—reduces the probability of charge carrier recombination.

In PC, the required charge carrier diffusion length is influenced by the distribution of active catalytic sites on the material. Uniform cocatalyst integration across the material surface is thus highly important. Depending on the system and particular metal, cocatalysts can also interact with excitons. Pt, for example, can act as a productive electron sink to drive charge separation.^21^ This generally occurs in systems with low activity however, and idealized systems have charge generation independent of cocatalysts. At higher concentrations, cocatalysts can also have negative effects, blocking light or facilitating exciton recombination,^22^ and so the loading amount should also be considered.

### Device Design

**Figure 3:**
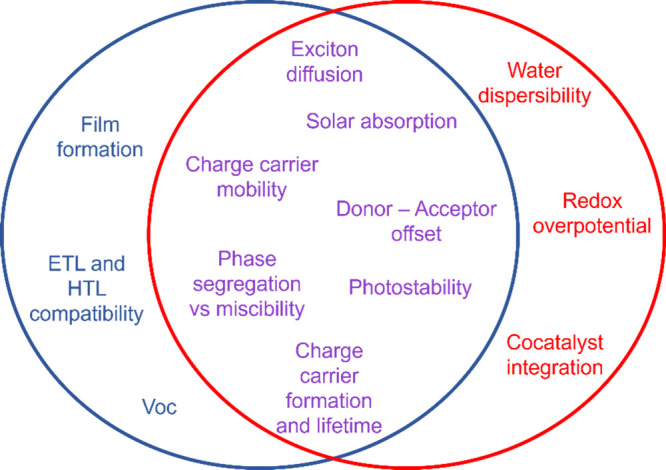
Shared (purple) and exclusive design considerations in OPV (blue) and organic PC (red). Acronyms used for electron transport layer (ETL) and hole transport layer (HTL).

While studies into film-based organic photocatalysts have been conducted,^23^ it is argued that the ideal device format for water splitting is a particle suspension.^11^ In such a system, no electrodes are required to collect charges, and therefore, each particle acts as a minidevice—catalysis happens on the surface of every particle—with associated efficiency benefits from the substantially smaller length scales needed for charge transport. This is particularly important in PC over PV. In the latter, charge transport is directed (through the small film thickness) by an internal electric field across the device. No such field is present in PC and thus the uniformly small dimensions of nanoparticles are preferred. *Water dispersibility* must therefore be added to the list of materials requirements for PC—a factor that most definitely does *not* apply to electrode-sandwiched, thin film-based PV. The shared and mutually exclusive considerations to OPV and organic PC design are summarized in Figure 3.

## Quantifying Efficiency in Solar Fuel Photocatalysts

One advantage of film devices in OPV is that multiple descriptors can be used to measure efficiency (photocurrent, fill factor, *V*_oc_) and from that, a picture of *why* one material is better than the other can be ascertained. The electronic characterization techniques for aqueous organic particle suspensions are significantly less developed. In solar fuels, often the only measured output is hydrogen production. Thus, deconvoluting the particular property that makes one material better than another can be difficult.

This issue is exacerbated by the lack of benchmarking currently used in the solar fuels literature. Along with independent verification laboratories, PV has standardized solar spectra and light intensities for measurements. If studies deviate from a 1.5 AM solar spectrum and 100 mW cm^–2^ (1 sun) irradiation, this is, typically, clearly stated and justified. As a less mature field of study, solar fuels have not yet adopted standardization at sufficient levels. Light intensity data is frequently absent for headline rate experiments, even in papers disclosing “new records”. While most experimental details indicate that some UV wavelengths have been excluded by filtration, the filters used to do this vary from 300 to 420 nm cut-offs. This is highly significant as wide-band-gap materials, typically the most active materials for OWS, can have orders of magnitude different rates across this range. Ideally, a standardized spectrum—as in PV—would allow easier comparison between systems. At the very least, publications should provide the spectrum and intensity data for the light source used in their experiments.

Light source variation is compounded by a variety of different reporting metrics for hydrogen evolution rate (HER). The most confusing among these is the tendency to provide rates that are normalized to the mass of the catalyst. This is a legacy from thermal catalysis where the rate is often directly proportional to the number of active sites. As has been discussed in great detail elsewhere,^24,25^ this is not the case for solar fuel research. Solar fuel photocatalysts, like OPV, should ultimately be judged on efficiency per area of solar irradiation. While material cost should of course be considered, there is little point in quantifying a material’s activity in unrealistic conditions. A nanoparticle photocatalyst may be able to give incredibly high mass-normalized rates at very low catalyst concentration, but unless that material can be concentrated sufficiently to utilize all solar energy incident on the reactor then it is not a feasible solar fuels photocatalyst. Mass-normalized rates may be useful in determining some inherent materials properties, but they ignore the fact that photocatalyst dispersion and whole-system light utilization are fundamental factors that should be considered in materials design.

External quantum efficiencies (EQEs) in PC are used somewhat interchangeably with apparent quantum yields (AQYs) and are measured at a specific wavelength. They are generally defined as the percentage of incident photons that results in a successful photocatalytic reaction, i.e., for hydrogen production where two electrons are required to form one product molecule, twice the HER divided by photon flux. When recorded across several wavelengths to give an “action spectrum”, these metrics are presented as a comparison between materials. This is undoubtedly an improvement on ill-defined hydrogen evolution rates, but the question of photon intensity still remains, thus undermining reliability. In PV, EQEs are also measured as a function of wavelength using monochromatic light, but for these measurements, a light bias is used. A realistic carrier concentration is generated using a background 100 mW cm^–2^ 1.5 AM spectrum (1 sun), and the EQE for each wavelength is then measured as a current change on top of this. This is a more challenging measurement to carry out for PC, as hydrogen evolution measured by gas chromatography is both significantly less sensitive and significantly slower per measurement than photocurrent. In reality, EQEs in PC are measured at the solar photon intensity of the particular wavelength, i.e., very low overall intensity and unrealistic charge carrier concentrations. Achieving an equivalent 100 cm^–2^ concentration of a single wavelength is challenging with typical set-ups and requires multiple high-power LEDs per wavelength. As such, it is important to continue to push for standardized testing set-ups for non-EQE measurements such that full-spectrum HERs can be compared.

To avoid confusing comparisons, the discussion below only compares HER rates within a single study or compares EQEs that use similar photon intensities. Unless otherwise stated, the systems below use a photodeposited Pt cocatalyst and an ascorbic acid electron donor.

## OPV Materials for Photocatalytic Hydrogen Production

**Figure 4:**
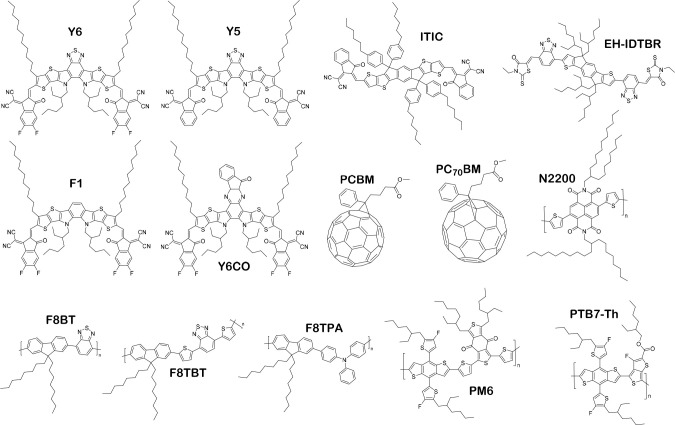
Chemical structures of OPV materials discussed herein.

After the 2009 discovery that graphitic carbon nitride—a carbon- and nitrogen-containing 2D polymer—could photocatalyze the production of hydrogen from water under visible light,^26^ there was a surge of interest in conjugated organic polymers for this application. Initial studies mostly focused on cross-linked materials.^27−30^ The linear polymers that were studied had no side chains attached to the backbone and so, like carbon nitride, were insoluble in organic solvents and thus unprocessable beyond simple particles.^31,32^ In 2016, however, Tian and co-workers showed that an early generation OPV acceptor, F8BT (Figure 4),^33^ was active for photocatalytic proton reduction.^34^ The solubilizing side chains on this material facilitated fabrication of aqueouse suspensions of polymer nanoparticles using nanoprecipitation with a PS-PEG-COOH surfactant. Follow-up studies focused on increasing planarization of the backbone and narrowing the optical gap to utilize more of the visible spectrum by introducing thiophene linkers on either side of the benzothiadiazole unit (F8TBT, Figure 4).^35^ As in photovoltaic devices,^36^ this leads to a significant improvement in performance with the HER increasing by a factor of more than 5. Similar studies with alternative acceptor polymers containing perylene diimides^37^ and more donor-type polymers, with carbazole^38^ or benzodithiophene,^37^ also showed activity for visible light-driven proton reduction. Over the past decade, the study of polymer acceptors for OPV has mostly, although not completely,^39^ been usurped by nonfullerene acceptors (NFAs), which now significantly outperform fullerene-based devices.^40^ Among these, the Y series of acceptors^41^ displays the highest efficiencies with power conversion efficiencies (PCEs) close to 20%.^42^ Typically, the fluorinated molecule, Y6, outperforms the nonhalogenated end group analogue, Y5 (both shown in Figure 4), in binary OPV devices due a combination of red-shifted absorption, closer and more slip-stacked π–π interactions, and reduced reorganization energies.^43^ However, when nanoparticles of Y6 and Y5 were recently tested for photocatalytic proton reduction,^44^ it was found that Y5 had a HER more than 14 times higher than Y6. The authors ascribe this primarily to formation of a longer lived triplet exciton in the Y5 material, but the increased driving force for proton reduction by the more shallow LUMO could also play a role. Clearly, the structure–property relationships derived from OPV do not always translate upon switching to a photocatalytic application.

Compared to non-OPV-derived, unprocessable, organic materials, which commonly show EQEs exceeding 10%,^45−48^ the efficiency of the above-mentioned materials for sacrificial hydrogen production is uniformly low. Even the state of the art Y series of NFAs does not have maximum EQEs that can exceed 1%.^44^ This is perhaps unsurprising when one takes into account that these materials are not designed to be single-semiconductor systems. The OPV equivalent to these photocatalysts would be a single-component photovoltaic device which, for organic materials, are at best^49^ capable of PCEs around 20 times less than their two-component, heterojunction analogues and in which most materials are completely incapable of generating charge-separated states.

While proton reduction under sacrificial conditions (i.e., with an easily oxidizable electron donor present to quench photogenerated holes) can rely on a reductive exciton quenching regime to generate electron polarons,^21^ overall water splitting is unlikely to proceed via such as mechanism due to the slow kinetics of water oxidation.^50^ A key concept of OPV is the ability to blend together p- and n-type semiconductors to form a heterojunction capable of separating strongly bound Frenkel-type excitons. The utilization of a donor–acceptor, two-semiconductor heterojunction was first applied to organic solar fuel photocatalysis in 2019.^51^ F8TPA and F8BT (Figure 4) polymer nanoparticles were studied individually for proton reduction before combining them in a blended material. Crucially, the authors showed that the blended nanoparticles had higher HERs than both the individual components and a physical mixture of the two types of nanoparticles. This rules out increased absorption as the cause of the higher performance and, along with fluorescence quenching experiments, indicates that the charge transfer from the donor to the acceptor within the nanoparticles is instead responsible.

The two materials used in this study represent first-generation organic semiconductors. They have significantly limited absorption ranges and lower PCEs than current state of the art materials. As such, the introduction of a highly efficient OPV blend combining the NFA, EH-IDTBR with a PTB7-Th donor polymer (Figure 4), represented an important finding for photocatalytic proton reduction.^52^ A novel aspect of these nanoparticles was their fabrication in a nanoemulsion, which is discussed in more detail in the Nanoemulsion Surfactant Effects section. The optimal blend of PTB7-Th:EH-IDTBR (3:7 by mass) had an intermixed heterojunction nanoparticle morphology and could produce hydrogen at more than 8 times the rate of either single-component nanoparticle. In comparison to literature materials, single-component wide-band-gap polymers have higher EQEs at wavelengths less than 500 nm. However, it appears that the charge separation properties of these heterojunction nanoparticles allow for a smaller overpotential for proton reduction than typically seen for single-component materials.^53^ This means that the narrow-band gap-semiconductors employed in this study can give unprecedented broad-spectrum activity, with EQEs of more than 6% at 700 nm. Another study investigated the same system and focused on the generation of charges using time-resolved microwave conductivity (TRMC).^54^ It was found that HER of nanoparticles across a range of donor:acceptor (D:A) mass ratios strongly correlated with the low-fluence TRMC signal strength (the product of charge carrier yield and mobility). The higher TRMC signal strength of the blended materials indicates that their high activity is indeed the result of an effective charge-separating heterojunction interface. The study concludes that even in the most efficient blends, hydrogen production is still limited by the yield of photogenerated charges, rather than by the catalytic rate at the interface. As the authors point out, this is particularly exciting for the organic materials chemist as it indicates that further study should focus on increasing charge carrier generation and reducing recombination rather than optimizing the metal cocatalyst.

To further understand the capability of the nanoemulsion technique, an investigation was conducted on of two of the most active blends from the OPV literature: one with a fullerene acceptor (PM6:PC_71_BM) and one with a NFA (PM6:Y6).^14^ Structures are shown in Figure 4. The latter blend showed an intermixed morphology similar to the first study on EH-IDTBR:PTB7-Th but with higher HERs, driven by the material’s higher absorbance at near-IR wavelengths. Interestingly, the PM6:PC_71_BM blend showed a more phase-separated core–shell structure that, at the 2:8 D:A mass ratio found to give optimal HER, had particles with sections of exposed core PC_71_BM in what was described as a “broken shell” morphology. The platinum nanoparticle cocatalyst photodeposits selectively on these exposed core sections, demonstrating why the lower acceptor loadings—where the PC_71_BM is fully surrounded by PM6 and has limited contact with the electrolyte—work less efficiently. The broken shell PM6:PC_71_BM nanoparticles showed 69% higher activity than the intermixed PM6:Y6 nanoparticles. This is particularly noteworthy given the blend has a significantly more blue-shifted absorption range. Comparing EQEs at 560 nm where both blends absorb strongly, the PC_71_BM blend has over 3 times the efficiency of the Y6 blend. To elucidate the reason behind this large difference, the excited state kinetics of the two nanoparticle blends were analyzed using transient absorption spectroscopy (TAS). In a *just water* suspension (without any electron or hole donors), both materials showed efficient and fast electron transfer from PM6 to the acceptor, resulting in formation of a strong PM6^+^ signal on a ∼1 ps time scale. Most significantly, both blends show a remarkable ∼4 s lifetime for the PM6^+^ species. Notably, the PM6:PC_71_BM particles showed a much larger accumulation of these long-lived states—approximately 3 times the signal amplitude—matching well with the observed EQE difference. This was suggested to be due to the more phase-segregated morphology of the PM6:PC_71_BM particles, which could help to prevent the recombination of charges. Addition of ascorbic acid to the nanoparticle dispersions resulted in complete quenching of the PM6^+^ signals. This is consistent with subsequent findings for the PTB7-Th:EH-IDTBR system^54^ and indicates that once formed, these hole polarons are easily accessible and transfer rapidly to the reductant. Unfortunately, the HOMO of PM6 is too shallow to drive water oxidation, but this study represents a milestone for organic photocatalysts; long-lived polaronic species have been observed on conjugated polymer and carbon nitride photocatalysts before, but the (in these cases) electron polarons only form upon addition of fast acting hole scavengers.^21,55^ Replication of these results in a similar system with a deeper lying donor HOMO and indeed integration of these materials into a Z-scheme with a separate OEP both represent exciting areas of study for overall water splitting.

Another work used a more standard nanoprecipitation fabrication for heterojunction nanoparticles and a high-throughput methodology, studying 5 polymer donors blended with 4 OPV-derived acceptors at various ratios, to give a library of 237 samples.^56^ The combinatorial strategy showed the broad scope of this approach; three of the four acceptors showed significantly improved HER in combination with three or more of the five donors. Particularly high activity was found for blends using PC_60_BM and ITIC acceptors (Figure 4) with the most active materials reaching EQEs of more than 3% at 600 nm.

Several other studies on nanoprecipitated polymer nanoparticles have investigated heterojunction systems;^57−59^ a study on a nanoparticle blend of F8TBT donor polymer and ITIC acceptor showed, similar to the nanoemulsion systems, that highly intermixed nanoparticle morphologies give efficient charge carrier generation but that this structure also leads to increased charge recombination and limits the further utilization of free charges.^57^ This is analogous to many OPV blends where the degree of donor–acceptor intermixing must be finely balanced, enough blending to give efficient charge separation but not so much that charge recombination becomes dominant.

**Figure 5:**
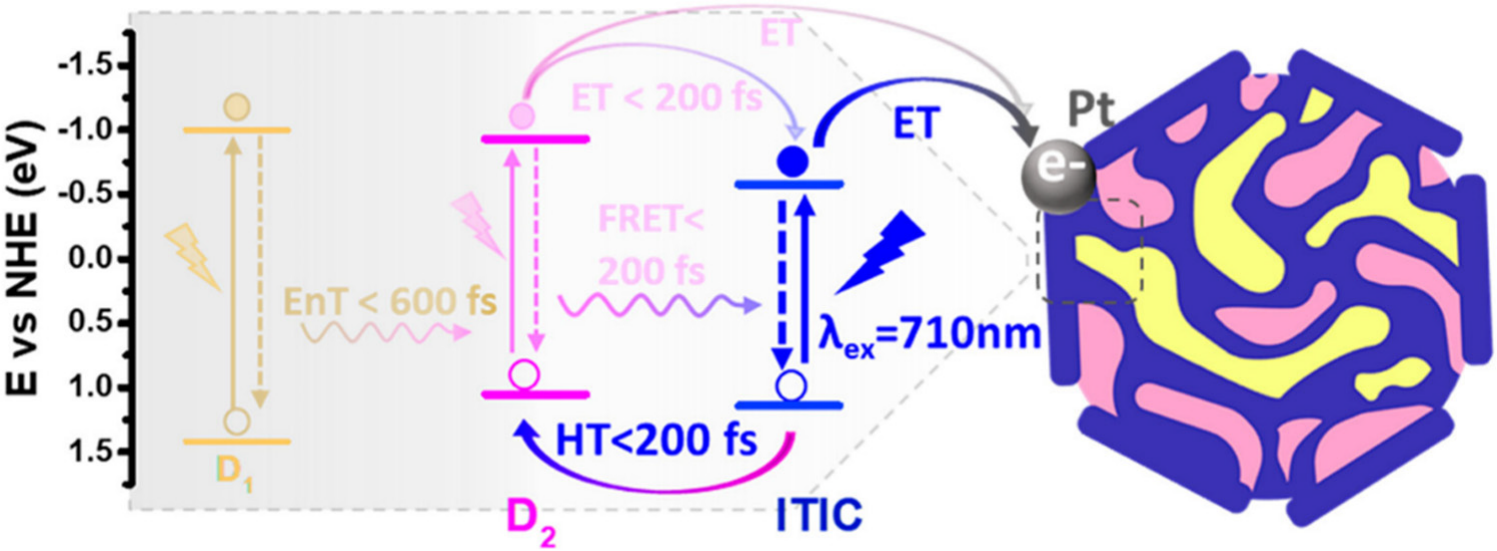
*E*nergy transfer mechanism and cartoon morphology of “panchromatic” ternary nanoparticles. D_1_ = F8BT, D_2_ = F8TBT. Adapted from ref (56). Copyright 2021 American Chemical Society.

In 2021, improved photocatalytic hydrogen evolution was demonstrated using a ternary blend system similar to those recently exploited in OPV.^59^ The D_1_:D_2_:A F8BT:F8TBT:ITIC nanoparticles were “panchromatic” with absorption efficiency above 90% across the entire visible range. The photocatalytic efficiency was also high (>4% EQEs) from 500 to 700 nm with an EQE max of 7.1% at 600 nm. Despite the rapid nucleation caused by the nanoprecipitation fabrication, TEM was employed to show crystalline regions of ITIC acceptor within the nanoparticles, which the authors suggest are crucial to high HER as they facilitate electron transfer to the Pt cocatalyst. Careful photophysical characterization by steady state fluorescence, TAS, and spectroelectrochemistry was used to identify the complex series of subpicosecond energy and charge transfers (shown in Figure 5) between the three components that give the nanoparticles this broad-spectrum efficiency. The development of ternary OPV design rules is somewhat limited by the significant variation in mechanism from blend to blend^60^—a difficulty that is also likely to affect ternary photocatalytic systems. However, the fact that a third component can be introduced to enhance the absorption efficiency without compromising the efficiency of the binary system through quenching or morphology disruption is highly promising for the use of ternary systems in photocatalytic hydrogen production.

## Modified OPV Materials for Photocatalytic Hydrogen Production

**Figure 6:**
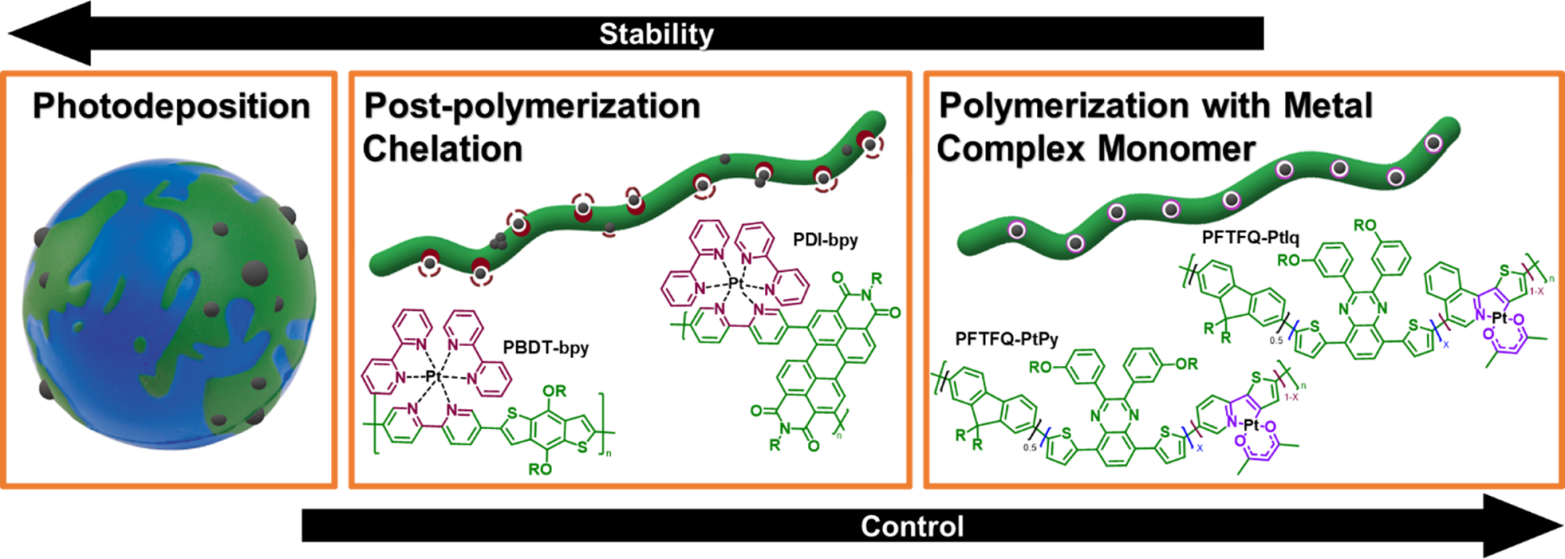
Methods of cocatalyst integration, and examples of chemical structures utilizing these.

The mechanism of OPV aims to transfer photogenerated charges to electrodes and relies on the internal bias of the heterojunction system and various electron and hole transport layers to do this. In contrast, photocatalytic water splitting, and indeed most solar fuel making reactions, typically rely on the transfer of photogenerated charges to one or more cocatalysts that act as active sites for redox. The semiconductor–cocatalyst–electrolyte interfaces are therefore crucial to efficiency but are frequently unoptimized in solar fuel systems. Most studies rely on photodeposition of metal nanoparticles (Figure 6, left) onto the semiconductor surface from aqueous platinum salts (for proton reduction) or cobalt salts (for water oxidation).^61−63^ This locates the cocatalysts at the appropriate hole or electron-rich sites of the material but offers little room for control and optimization. The easily modified structures of OPV materials means that metal chelating groups can be built in via molecular design (Figure 6, middle and right) in an attempt to control the amount, distribution, and size of the cocatalyst particles.

One strategy is to generate a metal complex monomer and copolymerize these with typical organic semiconductor monomers. For example, Pt(C^∧^N) chromophores can be chelated with an O^∧^O diketonate to generate cycloplatinated monomers PtPy and PtIq. A 2018 study added these into PFTFQ (Figure 6, right) at various loadings and tested the polymers for photocatalytic proton reduction. Maximum HERs were measured for polymers with 15 mol % platinum complex.^64^ This approach has also been expanded to a variety of benzo[1,2-c:4,5-*c*′]dithiophene-4,8-dione and benzo[*d*][1,2,3]triazole bearing OPV-type materials, and it has been demonstrated that the polymers with the integrated cycloplatinated monomer had higher HER than the noncycloplatinated materials with Pt instead added via photodeposition.^65^

Metal cocatalysts can also be added post polymerization if weaker coordinate bonding groups are used. Yu and co-workers demonstrated the addition of a cobalt cocatalyst by integration of chelating bipyridine monomers into PBDT- and PPDI-based polymers (Figure 6, middle).^37^ These materials are synthetically easier than making metal-complex monomers as metal is added via simple impregnation. However, this gives a less defined cocatalyst species where free metal salt and alternative chelating groups could play a role. This approach does open up the range of metal centers that can be introduced. Reductive photodeposition is limited to Pd and Pt, both expensive noble metals, while chelation can bind more earth-abundant transition metals, such as Fe and Co.

In 2023, the bridgehead of the Y6 core was modified to introduce a carbonyl-bearing “claw” which could coordinate a Pt cocatalyst.^15^ The resulting material, Y6CO (Figure 4), had increased photocatalytic activity compared to Y6 in both single-component nanoparticles and heterojunctions with PM6. Pt was added by photodeposition, and it was shown that Pt^0^ formation on the Y6CO nanoparticles was faster than that with Y6, with higher photodeposition yields on Y6CO observed by ICP, although only on a thin film. The deposition surface of a nanoparticle is very different to a film, not least because of the presence of surfactant (discussed in more detail later). TEM did show ∼1 nm Pt particles on the Y6CO nanoparticles, similar to previous studies on a number of different materials. Unfortunately, no high-resolution microscopy was conducted to compare the distribution or size of the Pt particles on systems with and without the σ–π "claw" anchor, and so the exact nature of this coordination and its effect on cocatalyst particles remains unknown. For example, whether the cocatalyst coordination results in single-atom Pt—as has been shown in various COF-based materials—or whether a small number of coordinating groups on one of the larger particles facilitates the higher activity remains a question. The authors suggest that the increase in catalytic activity is the result of a reduction in the d-band center of Y6CO–Pt^0^ and the associated lower H_2_ adsorption energy. This is based on calculations assuming a single-atom model, and so further microscopy studies would be useful to verify whether this is indeed the cocatalyst structure. Regardless of the exact cause, this material is undeniably an improvement on Y6. Heterojunction nanoparticles blending Y6CO with PM6 in a 7:3 mass ratio have remarkable activity with an EQE of over 10% for the first time at 800 nm.

A separate study employed F1 (Figure 4), an acceptor which replaces the thiadiazole bridgehead of the Y6 core with a phenyl bridgehead, and also observed increased photocatalytic activity.^66^ The design strategy in this case was to use a less polar group to reduce the reorganization energies of the fluorophore and therefore the rate of nonradiative decay. This was a successful strategy, with the F1 material showing a 66% increase in photoluminescence quantum yield to 9.3% and a calculated exciton diffusion length of 20 nm versus Y6’s 12 nm. However, these measurements, along with the morphological analysis, were conducted on spin-coated films of the NFAs. The assumption that these properties can be extended to nanoparticles formed under nanoemulsion conditions remains unproven, as solvent removal speeds and surfactant can often result in observable changes to NFA packing.^52^ The authors used electrochemical impedance spectroscopy to show the F1 nanoparticles also had improved charge transport compared to Y6 nanoparticles, and this along with the increased driving force provided by F1’s 0.2 eV more shallow LUMO also likely contribute to the increased HER observed for this material. The alternately more polar^15^ or less polar^66^ bridgehead substitution employed by these two studies perfectly illustrates the challenge of developing organic photocatalyst materials; catering to one property can often involve molecular design strategies that directly hinder another (Figure 2).

Given a significant difference between OPV and photocatalysis is the aqueous environment of the latter application, one obvious strategy to convert good OPV materials into good photocatalysts is to swap the alkyl side chains for more hydrophilic glycol chains. This was first achieved for photocatalytic hydrogen production in 2016 with little difference in photocatalytic activity between the glycolated or the alkylated BDT-bpy (Figure 5, middle) polymers developed.^37^ However, since then, multiple studies have shown large positive effects of glycolating OPV-style materials for photocatalysis;^23,67,68^ notably, when copolymerized with fluorinated benzothiadiazole, glycolated benzodithiophene shows a HER that is 90 times higher than that of an alkylated analogue.^67^ These materials were tested as simple powder suspensions in water, and to a large extent the increase in HER is a function of increased dispersibility, giving an increased catalytically active surface area and less wasted light absorption by the interior of large particles.^69^ With the advent of nanoemulsion techniques to generate small dispersible nanoparticles of even hydrophobic materials, this factor has arguably become less significant. However, glycolation has several other notable effects; first, it has been shown using XPS that glycolated polymers have a stronger interaction with Pt, thought to aid in energy transfer from polymer to cocatalyst.^67^ Second, glycolated materials show increased swellability in a water environment, i.e., integration of water into the polymer particle itself.^23^ This is crucial for the transfer of charges from the polymer and cocatalyst to the electrolyte-based catalytic reagents. Glycolated materials have been shown, via electrochemical impedance spectroscopy, to have increased charge carrier mobility in some cases,^67^ but it has also been found that glycol side chains cannot pack in an all-trans, ordered conformation, in comparison to alkyl chains, and often this increased disorder leads to lower mobility.^70^ Most interesting is the effect of glycolation on charge carrier generation and lifetime; glycolation has been show to give increased charge carrier generation in terms of photocurrent response^67^ and larger ΔOD values in transient absorption spectroscopy, both in reductive quenching regimes^23^ and without hole scavenger.^68^ Notably, it has been found that some single-component glycolated materials can form long-lived charge-separated states upon excitation.^68^ This is thought to be due to the increased relative permittivity inside the nanoparticles. The fact that both short-timescale exciton/charge transfer state separation processes and longer-timescale charge recombination appear to be affected suggests that the polarizable ether groups of the glycol may not be solely responsible for this change.^71^ Instead, the chain may also encourage uptake of water into the nanoparticles. This would increase the *high-frequency* relative permittivity in particular, reducing coulombic attraction between bound states and explaining the altered picoseconds to nanoseconds behavior. Interestingly, it has also been found that the charge carrier lifetime in aqueous suspensions of nonglycolated heterojunction nanoparticles is significantly higher than that of dry, spin-coated heterojunction films.^14^ Given the level of optimization that goes into OPV blend films, this is surprising. It could suggest that the aqueous environment surrounding or within the nanoparticles, perhaps along with the ionic surfactant, significantly alters the relative permittivity experienced by charge carriers and increases their lifetime. This effect would likely be applicable across a range of donor–acceptor blends and suggests that these materials could potentially be preferentially suited to PC, even in comparison to PV.

The degree to which the electrolyte penetrates into nanoemulsion-derived nanoparticles is a question that warrants further investigation. Electrolyte penetration into porous organic polymers is a key design principle in many organic solar fuels photocatalysts and has been shown to give a high catalytically active surface area and improved mass transfer.^45,72^ However, the picture with regards to linear, nonporous polymers is less clear. OPV blends typically form films with close-packed polymer and molecular units which, along with their hydrophobicity, would not necessarily facilitate water uptake. Whether this would even be desirable is also questionable. Significant swelling of the polymer/NFA blends could disrupt the morphology, thus reducing the charge transport properties of the materials as well as reducing the charge-separating interfacial area between the donor and the acceptor. In principle, a porous, donor–acceptor charge-separating heterojunction which incorporates water into a rigid structure, without disrupting packing, could overcome this, for example, a covalent–organic framework like structure.^73^ For flexible OPV-type structures, an alternative tactic is to increase the catalytically active nanoparticle surface area by reducing the particle size. Nanoemulsion fabrication typically gives sub 100 nm particles, but these are still significantly above the ∼10 nm exciton diffusion lengths typical of these materials. Reducing the particle size to these length scales could reduce the proportion of photogenerated charges that do not reach the particle surface.

## Nanoemulsion Surfactant Effects

As mentioned previously, the use of nanoemulsion nanoparticle fabrication has significantly expanded the capability of OPV materials in photocatalytic hydrogen production. Previously, nanoparticles could only be fabricated through nanoprecipitation methods which can lead to dispersions with low stability with respect to particle aggregation^34^ and limit materials to those that are soluble in low boiling point, water-miscible solvents such as THF. The highest performing OPV semiconductors are typically only soluble in chlorinated solvents such as chloroform, where the interplay of solvent, polymer, and molecules during film drying is crucial in forming efficient morphologies for charge separation. The ability to transfer this into a nanoparticle system is crucial in fully exploiting preexisting OPV materials and knowhow.

A surfactant is required to stabilize the two-phase chloroform–water nanoemulsion during semiconductor crystallization/precipitation and to stabilize the nanoparticles against flocculation in the aqueous suspension. Even if excess surfactant can be removed post fabrication, a proportion will remain, with the hydrophobic head preferentially inserted in the organic semiconductor domains. Study of the surfactant effects on nanoparticle formation and in photocatalysis are thus highly important. 2-(3-Thienyl)ethyloxybutylsulfonate sodium salt (TEBS) was first employed in the nanoemulsion fabrication of OPV blend nanoparticles in 2019, although not for photocatalytic applications.^74^ The study showed that using TEBS resulted in P3HT:PCBM nanoparticles with an intermixed structure, while the more standard surfactant, sodium dodecyl sulfate (SDS), resulted in core–shell structures. The same behavior in PTB7-Th:EH-IDTBR blended nanoparticles was found, which was ascribed to the similar chloroform/water interfacial tension in the presence of TEBS when the chloroform phases contained EH-IDTBR versus PTB7-Th. The longer chain, nonaromatic SDS surfactant has a higher affinity for PTB7-Th, and so it is energetically favorable for the donor phase to segregate at the water-chloroform interface during drying. Moving from SDS to TEBS, i.e., from core–shell to intermixed donor–acceptor morphology, was accompanied by an order of magnitude increase in HER, a phenomenon which was suggested to result from (1) the increased donor–acceptor charge separation interface and (2) the ability of the acceptor phase to interact with the electrolyte (as well as the donor) and be exposed for photodeposition of the Pt cocatalyst.^52^

A follow-up study focused on the choice of either SDS or TEBS surfactants on nanoparticles of Y6.^75^ Interestingly, these materials also showed an order of magnitude increase in HER on moving from SDS to TEBS. Clearly, intermixed and core–shell morphologies are not possible in this single-component system, and instead, it is argued that the difference in HER for TEBS- and SDS-stabilized Y6 is primarily due to inefficient photodeposition of Pt on the surface of the latter particles. They provide evidence of this through TEM and ICP, amounting to a 40% difference in Pt loading. The SDS-stabilized nanoparticles however still contained 0.73 wt % Pt. The difference in HER between the two materials is more than 2500%. Undoubtedly, the lower number of active sites for proton reduction results in less efficient utilization of photoexcited states and lower hydrogen production. However, given the mismatch between Pt loading and HER difference and that the mechanism of Pt deposition is a reductive charge transfer from photoexcited semiconductor to Pt, it could be argued that poor Pt deposition yields are more of a symptom of poor photocatalytic ability than the root cause. In earlier studies, different two-component nanoparticle morphologies were investigated, but the surfactant choice was also found to dramatically alter the photocatalytic activity of the single-component nanoparticles.^52^ For example, EH-IDTBR nanoparticles stabilized with an SDS surfactant had very low HER, producing less than 2 μmol of hydrogen over the 16 h testing period, while EH-IDTBR nanoparticles stabilized with a TEBS surfactant tested under identical conditions produced over 100 μmol. TEM analysis showed that the materials had somewhat similar Pt photodeposition, but there were visible changes to the morphology of the nanoparticles; both particles were crystalline with a lattice spacing of 1.6 nm, but the SDS formed particles mostly consisted of single crystals of EH-IDTBR, while the TEBS formed particles were polycrystalline. We attributed these changes to the higher chloroform/water interfacial tension in the presence of TEBS versus SDS and hence more rapid nucleation of EH-IDTBR in the former system. How exactly this morphology effects the hydrogen evolution rate, however, is still something that warrants further investigation; a difference in the UV–vis spectra of TEBS- versus SDS-stabilized Y6 nanoparticles has been shown, which also indicates differential molecular packing. Specifically, the magnitude of the ∼820 nm absorption peak is generally less prominent in the nanoparticles than in Y6 films but the TEBS stabilised particles retain higher absorption in this region than the SDS stabilised. This 820 nm feature is known to be the result of a specific J-type aggregation mode that many have been ascribed as a contributory factor to Y6’s high efficiency.^76^ It is possible that the differing nucleation conditions provided by each surfactant during nanoemulsion results in different Y6 packing, different photophysical and electronic properties, and thus differing HER. This would be consistent with studies on EH-IDTBR, although admittedly TAS analysis into the excited state kinetics of the Y6 nanoparticles appears to show only small differences between the TEBS- and SDS-stabilized Y6.^75^

Also of note in terms of surfactant effects is the aforementioned TRMC measurements on PTB7-Th: EH-IDTBR.^54^ Similarly to TAS studies on PM6:Y6,^14^ these showed considerably higher charge accumulation in nanoemulsion-derived nanoparticles than in spin-coated films, but unlike the TAS measurements, the nanoparticles were not under hydrated conditions. Instead, the nanoparticles, with surfactant, were suspended in a dry cellulose matrix. This removed any potential charge-stabilizing effects of water, but a 3-fold difference in charge accumulation in nanoparticles in comparison to a film was still observed. The NPs gave consistently higher signals across multiple D:A ratios, suggesting that a differing interface morphology may not be the sole cause of this improvement. We speculate that the surfactant could be aiding in charge carrier generation or even inhibiting recombination.

## Conclusions and Outlook

The most obvious way to address the question in the title of this paper is to look at the raw HER data of OPV materials. Under this lens, OPV materials are a good, if not perfect, match for solar fuel applications; they may have lower efficiencies for hydrogen production at near-UV wavelengths than equivalent carbon nitride or COF materials, but their broad-spectrum EQEs are unparalleled.^15,59^ The scope of this suitability also seems to be quite wide; the studies discussed above represent a range of state of the art donor and acceptor materials. Similarly, while OPV materials have yet to be shown to be capable of overall water splitting and indeed have not been used in a Z-scheme system, they have been shown to be more able to form accessible (non-trapped), long-lived charge-separated states in water than any other organic materials.^14^

Another way to assess the titular question is to consider whether the huge library of knowledge associated with OPV, in terms of design principles and structure–property relationships, can be used in photocatalytic applications. Here, the answer is less clear. There certainly appears to be scenarios where, at first glance, photocatalytic activity trends in opposition to PCE. For example, PM6:Y6 nanoparticles show lower single-wavelength photocatalytic EQEs than PM6:PCBM^14^—in direct contrast to the PV PCEs. However, when one considers the overall broad-spectrum HER at equal Pt loadings, the difference between the two materials is much smaller; the metric of activity used for comparison is clearly important. In addition, the broken shell morphology of the PCBM blends appears to be a more optimal structure for reducing charge carrier recombination. Blend morphology control then is perhaps one area that will require separate optimization to OPV applications. Equally, the low HER of Y6 compared to Y5 and F1 particles in opposition to their blend PCEs is perhaps an unfair comparison. The use of a single-component material with a sacrificial electron donor completely removes the need for charge separation by the material. These tests are not a good analogue for PV, and while arguably helpful for determining individual semiconductor properties, they are also not a perfect analogue for photocatalytic hydrogen production in heterojunction systems aiming to move away from a reductive quenching regime. It would be interesting to see whether the exciton lifetime-induced differences in HER in these acceptors persist in heterojunction blends where excitons should instead be rapidly separated into charged states.

### Cocatalyst Integration

As discussed, there are already several examples of modification to the chemical structures of OPV materials to optimize them for photocatalysis. These show necessary promise in cocatalyst integration, which is a key challenge if OPV materials are to compete with porous materials such as COFs that have optimized structures for molecular catalyst,^77^ cluster,^78^ or single-atom integration.^79^ This will only become more important as systems aim for integration of oxidation cocatalysts (in combination with reduction cocatalysts) to allow for overall water splitting or proton reduction coupled with oxidation of reversible redox shuttles for Z-scheme systems.

### Stability

A crucial consideration for all organic photocatalysts, whether or not they are OPV derived, is stability in hydrogen-producing conditions. At best, materials are currently tested for 120 h, and even under these relatively short time scales, some reduction in activity is observed.^59^ Further studies are needed to determine and improve the long-term stability of heterojunction nanoparticles under aqueous conditions. As with OPV, the physical stability of the blend, including factors such as retention of crystallinity and resistance to leeching, will need to be investigated, as well as the stability of the nanoparticles in suspension. The molecular structures of organic semiconductors may also have inherent instabilities to pH, for example, and should be examined in more detail. Given the known issues with some OPV functional groups and reactive oxygen species, it would also be prescient to study the photochemical stability of these materials in the presence of oxygen. Z-schemes with solution-based redox shuttles could enable hydrogen evolution to occur under separate anaerobic conditions to oxygen evolution, but single-reactor systems are thought to be more promising^11^ if stability to oxygen is not an issue.

### CO_2_ Reduction

This paper has exclusively discussed solar fuels in the context of proton reduction to hydrogen. Photocatalytic carbon dioxide reduction to value-added products is also a burgeoning field^80−82^ but is not considered here due to a lack of literature examples using OPV materials. The reason behind this lack of uptake is not completely clear but two possibilities are (1) that CO_2_ reduction is typically most efficient at high pH, so the ∼4 eV LUMO of most OPV acceptors would give limited driving force under these conditions, or (2) that CO_2_ reduction is typically more efficient in dispersions that have a cosolvent in addition to water (due to both proton reduction competition and CO_2_ solubility) and the addition of cosolvents may cause aggregation of surfactant-stabilized aqueous nanoparticle dispersions.^83^ CO_2_ reduction to CO is, like proton reduction to hydrogen, a 2-electron process. However, accessing higher order reduction products, such as methanol, requires 6 electrons. The kinetics are thus very slow and would likely require specialized cocatalysts, such as those used for CO_2_ electroreduction.^84^ None of these problems would appear to be insurmountable with the correct FMO tuning, surfactant choice, and cocatalyst, so we believe heterojunction nanoparticles still also represent exciting possible candidates for CO_2_ reduction.

### Potential Candidate Materials

The rise of nanoemulsion fabrication for heterojunction nanoparticles means that essentially any OPV blend can now be investigated for photocatalysis. As stated initially, the first point of consideration when choosing candidates should be the absolute energy levels of the material and whether these have sufficient overpotential to drive the redox reactions in question. Given this and the general trend that high PCE blends translate to highly active heterojunction nanoparticles for hydrogen production, where else should we look? As mentioned, the −5.1 eV HOMO of PM6 is too shallow to drive water oxidation or even most reversible redox shuttles used in Z-schemes.^85^ Polymers with a deeper HOMO than PM6 are therefore desirable for the generation of photocatalytic nanoparticles with even higher efficiency for proton reduction and significantly more oxidative driving force.

Overall, the field of OPV has much to offer the solar fuel chemist. So long as care is taken to consider the different requirements of the two applications, the library of donor and acceptor semiconductors and the knowledge of their interaction is a veritable gold mine ready to be transferred into heterojunction nanoparticles. Blends taken directly or derived from OPV are likely to be crucial in the move away from sacrificial hydrogen production and toward unassisted overall water splitting. Inorganic OWS systems have shown exciting advances in recent years,^86^ and if organic materials are to be viable additions to these systems, this move needs to happen soon.

## Abbreviations

PV: photovoltaics
PC: photocatalysis
OPV: organic photovoltaics
IP: ionization potential
EA: electron affinity
HOMO: highest occupied molecular orbital
LUMO: lowest unoccupied molecular orbital
*V*_OC_: open circuit voltage
PCE: power conversion efficiency
FMO: frontier molecular orbital
SHE: standard hydrogen electrode
HEP: hydrogen evolution photocatalyst
OEP: oxygen evolution photocatalyst
D:A: donor:acceptor
ETL: electron transport layer
HTL: hole transport layer
OWS: overall water splitting
EQE: external quantum efficiency
AQY: apparent quantum yield
HER: hydrogen evolution rate
NFA: nonfullerene acceptor
TAS: transient absorption spectroscopy
TRMC: time-resolved microwave conductivity
TEBS: 2-(3-thienyl)ethyloxybutylsulfonate sodium salt
SDS: sodium dodecyl sulfate
PSMA: polymerized small molecule acceptor
